# Evaluating the Sensitivity of *Mycobacterium tuberculosis* to Biotin Deprivation Using Regulated Gene Expression

**DOI:** 10.1371/journal.ppat.1002264

**Published:** 2011-09-29

**Authors:** Sae Woong Park, Marcus Klotzsche, Daniel J. Wilson, Helena I. Boshoff, Hyungjin Eoh, Ujjini Manjunatha, Antje Blumenthal, Kyu Rhee, Clifton E. Barry, Courtney C. Aldrich, Sabine Ehrt, Dirk Schnappinger

**Affiliations:** 1 Department of Microbiology and Immunology, Weill Cornell Medical College, New York, New York, United States of America; 2 Center for Drug Design, University of Minnesota, Minneapolis, Minnesota, United States of America; 3 Tuberculosis Research Section, National Institute of Allergy and Infectious Diseases, Bethesda, Maryland, United States of America; 4 Department of Medicine, Weill Cornell Medical College, New York, New York, United States of America; 5 Novartis Institute for Tropical Diseases, Singapore; McGill University, Canada

## Abstract

In the search for new drug targets, we evaluated the biotin synthetic pathway of *Mycobacterium tuberculosis (Mtb)* and constructed an *Mtb* mutant lacking the biotin biosynthetic enzyme 7,8-diaminopelargonic acid synthase, BioA. In biotin-free synthetic media, *ΔbioA* did not produce wild-type levels of biotinylated proteins, and therefore did not grow and lost viability. *ΔbioA* was also unable to establish infection in mice. Conditionally-regulated knockdown strains of *Mtb* similarly exhibited impaired bacterial growth and viability *in vitro* and in mice, irrespective of the timing of transcriptional silencing. Biochemical studies further showed that BioA activity has to be reduced by approximately 99% to prevent growth. These studies thus establish that *de novo* biotin synthesis is essential for *Mtb* to establish and maintain a chronic infection in a murine model of TB. Moreover, these studies provide an experimental strategy to systematically rank the *in vivo* value of potential drug targets in *Mtb* and other pathogens.

## Author Summary

We evaluated the biotin synthetic pathway of *Mycobacterium tuberculosis* (*Mtb*) as a new drug target by first generating an *Mtb* deletion mutant, *ΔbioA*, in which the biotin biosynthetic enzyme 7,8-diaminopelargonic acid synthase (BioA) has been inactivated. This mutant grew in the presence of biotin or *des*-thiobiotin, but not with an intermediate of the biotin biosynthesis pathway that requires BioA to be converted into biotin. Without exogenous biotin or *des*-thiobiotin, *ΔbioA,* was unable to produce biotinylated proteins, which are required for the biosynthesis of fatty acids, and thus died in biotin-free media. Using a regulatable promoter and different ribosome binding sequences we next constructed tightly controlled TetON mutants, in which expression of BioA could be induced with tetracyclines, but was inhibited in their absence. Characterization of these mutants during infections demonstrated that *de novo* biotin synthesis is not only required to establish infections but also to maintain bacterial persistence. Inhibition of BioA or other enzymes of the biotin biosynthesis pathways could thus be used to kill *Mtb* during both acute and chronic infections. Biochemical and immunological analyses of different *Mtb* mutants indicate that drugs targeting BioA would have to inactive approximately 99% of its activity to be effective.

## Introduction

*Mtb* causes approximately 8 million new cases of active tuberculosis (TB) and 2 million deaths each year [1]. Efforts to combat the TB pandemic have been hampered by the emergence of drug resistant strains of *Mtb* [2]. TB drug development thus represents a major area of unmet medical need. *Mtb* is shielded from the environment by a complex envelope that consists of an inner membrane, a periplasmic space, an outer membrane and a loosely attached capsule [3]–[5]. Isoniazid (INH), ethionamide (ETH) and ethambutol (EMB) constitute three anti-tuberculosis drugs that specifically inhibit synthesis of this envelope and validate cell envelope biosynthesis as a target pathway in *Mtb*.

**Figure 1:**
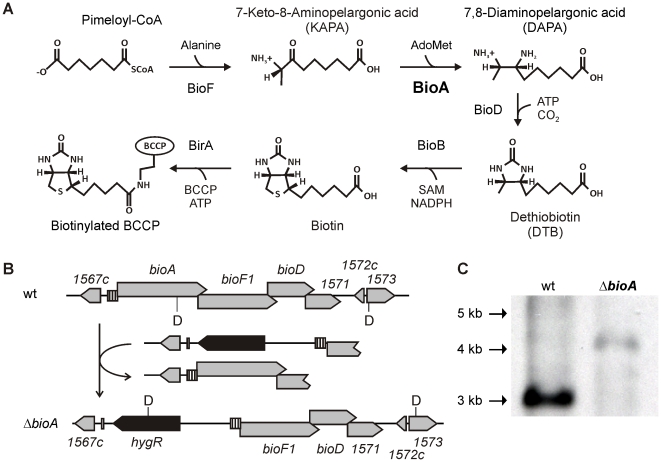
Biotin synthesis pathway and construction of *Mtb ΔbioA*. (A) Shown are the reactions catalyzed by KAPA synthase (BioF), DAPA synthase (BioA), DTB synthase (BioD) and biotin synthase (BioB), which convert pimeloyl-CoA to biotin, and biotin ligase, which attaches the cofactor to biotin-dependent enzymes. (B) The upper panel displays the genetic organization of the *bioA* region in wt *Mtb*; the lower panel displays that of *ΔbioA*. Gray boxes represent open reading frames of the genes specified above or below, the striped boxes mark the putative *bioA* promoter, and the black boxes represent hygR. The localization of recognition sites for the restriction endonuclease *Dra*III are labeled “D”. (C) Southern blot of *Dra*III-digested genomic DNA from wt *Mtb* and *ΔbioA*. The expected sizes for the *Dra*III fragments hybridizing to the *bioF1*-derived probe that was used in this blot are 3068 bp for wt and 4021 bp for *ΔbioA*.

Biotin is an essential cofactor required for synthesis of the fatty acid component of *Mtb*'*s* cell envelope [6]–[10] and is synthesized from pimeloyl-CoA via a pathway consisting of four enzymes, BioF, BioA, BioD and BioB (Figure 1A) [11]. Bioactivity of biotin further requires covalent attachment to an enzyme, via a biotin ligase [12]. While the source of pimeloyl-CoA in *Mtb* is unknown [13], mammalian cells lack the enzymes to synthesize biotin *de novo* and must acquire it from external sources. Based on this presumed essentiality and intrinsic bacterial specificity, we sought to validate biotin biosynthesis as a potential target for the development of new antibiotics.

That efficient inhibition of biotin synthesis by a small molecule can be achieved was demonstrated by the ability of amiclenomycin, a natural product inhibitor of BioA isolated from *Streptomyces lavendulae* [14], to prevent *in vitro* growth of *Mtb* in media lacking exogenous biotin [15]. Treatment of mice with amiclenomycin had no impact on infection with *Mtb*, which was interpreted as evidence that *Mtb* does not depend on biotin synthesis during infection and is instead able to scavenge this cofactor from the host [16]. However, the lack of pharmacokinetic data showing that the organism was, in fact, exposed to the drug in these experiments temper this conclusion. In contrast, genome wide mutagenesis studies identified transposon insertion mutants of biotin synthesis genes as among the most highly attenuated mutants of *Mtb* observed in mice [17], suggesting that *Mtb* cannot access exogenous biotin in mice. Here, we constructed genetically defined *Mtb bioA* mutants to determine the consequences of inhibiting biotin synthesis on growth and survival of *Mtb in vitro* and during acute and chronic mouse infections.

## Results

### BioA is required for protein biotinylation, and therefore the growth and survival of *Mtb* without exogenous biotin

The *Mtb bioA* gene is located upstream of *bioF1*, *bioD*, and a gene of unknown function, *rv1571* (Figure 1B). The start codons of *bioF*, *bioD*, and *rv1571* are each positioned directly upstream of the stop codons of their respective upstream genes. This overlapping genetic organization suggests that both transcription and translation of *bioA*, *bioF1*, *bioD* and *rv1571* mRNAs are coupled. We therefore generated a knockout cassette consisting of a hygromycin resistance gene flanked by 451 bp of *rv1567c* and its putative promoter on one end, and the putative *bioA* promoter followed by the first 723 bp of *bioF1* on the other (Figure 1B). Integration of this cassette into the genome of *Mtb* H37Rv deleted *bioA* and placed the DNA fragment predicted to contain the promoter and the translational initiation site of *bioA* upstream of *bioF1*. Creation of this deletion mutant, Δ*bioA*, was confirmed by Southern Blot (Figure 1C).

**Figure 2:**
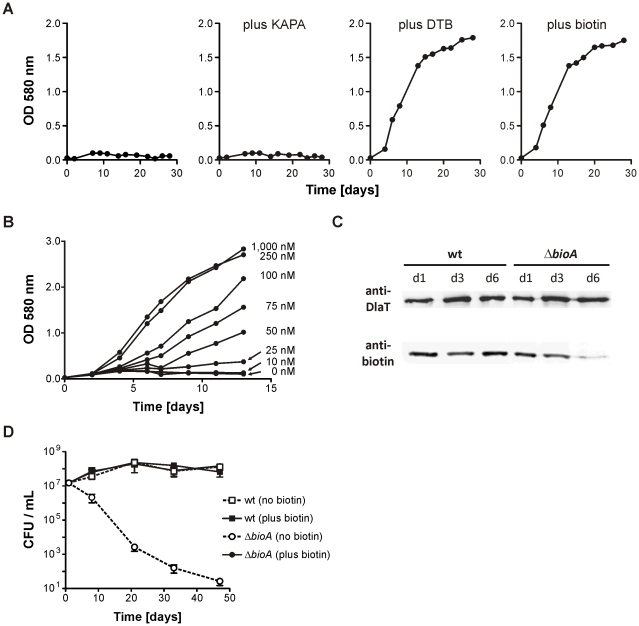
*In vitro* characterization of *Mtb ΔbioA*. (A) Growth of *ΔbioA* in Sauton's medium (left) or Sauton's medium supplemented with KAPA, DTB or biotin. Data are from individual cultures and representative of at least two independent experiments. (B)Growth of *ΔbioA* in Sauton's medium with varying concentrations of biotin. Data are from individual cultures and representative of at least two independent experiments. (C) Immunoblots performed with both an anti-biotin antiserum and rabbit serum recognizing *Mtb*'s dihydrolipoamide acyltransferase (DlaT). The lower panel shows the signal from the anti-biotin antibody; the upper panel shows the DlaT signal, which served as the loading control. Protein extracts were prepared after the indicated days of cultivation in biotin-free Sauton's medium. Data are from individual cultures and representative of at least two independent experiments. (D) Survival of wt and *ΔbioA* in Sauton's media with and without biotin. Data are averages from triplicate cultures and representative of at least two independent experiments. Error bars indicate standard deviations.

As expected, *ΔbioA* did not grow in liquid media without added biotin but could be rescued with exogenous biotin or *des*-thiobiotin (DTB), the substrate of the biotin synthase, BioB (Figure 2A). No rescue occurred with 7-keto-8-aminopelargonic acid (KAPA), the substrate of BioA. *ΔbioA* growth was dependent on the concentration of biotin in the media (Figure 2B); with little to no growth with exogenous biotin concentrations below 25 nM and wild-type (wt) levels of growth above 250 nM biotin. Anti-biotin immunoblotting similarly revealed a selective loss of immunoreactivity corresponding to a single protein in *ΔbioA,* but not wt *Mtb*, following 6 days of biotin starvation (Figure 2C). The electrophoretic mobility and anti-biotin immunoreactivity of this protein suggests that it corresponds to AccA3, one of three proteins predicted to be biotinylated in Mtb [6], [18].

We also assessed the impact of biotin starvation on *ΔbioA* viability by enumerating the colony forming units (CFUs) recovered after incubation for various time in Sauton's medium without biotin. *ΔbioA* CFUs decreased from 10^7^ CFUs per ml to less than 10^2^ CFUs per ml over a period of 50 days (Figure 2D). In the same medium, the CFUs of wt *Mtb* increased during the first two weeks and remained stable thereafter. CFUs recovered for wt and *ΔbioA* from biotin-containing Sauton's medium were almost identical. These experiments thus demonstrate that selective deletion of *bioA* caused a specific defect in *Mtb*'s biotin biosynthesis pathway as reported by biotin-dependent changes in protein biotinylation, growth and survival of *ΔbioA* but not wt *Mtb*.

### *BioA* is required for *Mtb* to establish acute infection in mice

*Mtb* mutants harboring transposon insertions in biotin synthesis genes were among the most strongly attenuated during infection of mouse spleens by pooled mutants [17]. We sought to extend these studies by measuring the extent to which *Mtb* depends on *de novo* biotin biosynthesis for growth in mouse lungs during acute, single-strain infection and for persistence in the chronic phase of infection. Whereas *ΔbioA* could address the first question, a mutant that enabled conditional silencing of *bioA* during the chronic phase of infection was required to answer the second. We therefore constructed a tetracycline repressor (TetR)-regulated mutant in which transcription of *bioA* can be induced with either anhydrotetracycline (atc) or doxycycline (doxy), but was inhibited in their absence. To achieve this, we cloned the *bioA* gene downstream of the TetR-controlled promoter P_myc1_ *tetO* [19] into a plasmid, which also contained a *tetR* whose codon usage was adapted to improve expression in mycobacteria [20]. Integration of the resulting plasmid, pGMCK-T2M1-*bioA*, into the attachment site of the phage L5 (attL5) yielded *Mtb bioA* TetON-1.

**Figure 3:**
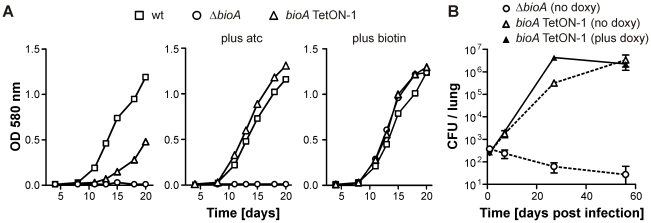
Growth of wt *Mtb*, *ΔbioA*, and *bioA* TetON-1 in liquid media and mice. (A) Growth of wt, *ΔbioA*, and *bioA* TetON-1 in Sauton's media, Sauton's media with atc or Sauton's media with biotin. Squares, circles, and triangles represent data for wt, *ΔbioA*, and *bioA* TetON-1, respectively. Data are from individual cultures and representative of several independent experiments. (B)Growth of *ΔbioA* and *bioA* TetON-1 in mouse lungs. Circles represent data for *ΔbioA*, triangles represent data for *bioA* TetON-1. Doxy was either given from day 1 of the infection or not at all. Data displayed by open symbols and dotted lines are from doxy-free mice, closed symbols represent data from doxy-fed mice. Data are averages from four mice per group; error bars represent the standard error of the mean.

Growth of *bioA* TetON-1 in biotin-free medium was indistinguishable from that of wt *Mtb* in the presence of atc, but reduced, though not abolished as observed for *ΔbioA*, in its absence (Figure 3A). Growth of wt, Δ*bioA*, and *bioA* TetON-1 in biotin-containing media, by contrast, was indistinguishable. Following aerosol infection, *ΔbioA* did not grow in mouse lungs. The *bioA* TetON-1 mutant grew slightly more slowly in mice not receiving doxy than in mice that were fed doxy but it reached a similar final bacterial load in lungs with and without doxy (Figure 3B). *In vitro* growth analyses confirmed that *bioA* TetON-1 bacteria recovered from mouse lungs at day 56 post-infection were still TetR regulated and did not represent suppressor mutants (not shown). In contrast to the muted phenotype of *bioA* TetON-1, *ΔbioA* was strongly attenuated and no or few CFUs were recovered 56 days post infections (Figure 3B). *BioA* is thus required for *Mtb* to establish an acute infection in mouse lungs.

### Construction of tightly controlled *Mtb bioA*-TetON mutants

**Figure 4:**
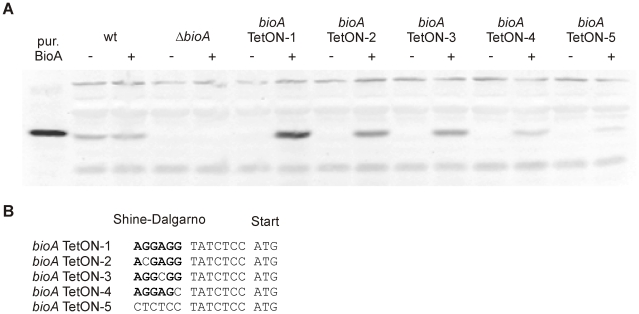
BioA protein levels of wt *Mtb*, *ΔbioA*, and *bioA* TetON mutants. (A) Immunoblots performed with anti-BioA antiserum using total protein extracts prepared from cultures grown with biotin. The plus and minus signs indicate presence or absence of atc in the culture medium. The first lane on the left contained purified BioA. (B) The putative Shine-Dalgarno sequence of *bioA* TetON-1 is shown in bold. The mutations that were introduced and distinguish the different TetON mutants are shown below. “ATG” indicates the start of the *bioA*.

To compare BioA protein levels of *bioA* TetON-1 with those of wt and *ΔbioA*, we grew them (with biotin) in the presence and absence of atc and analyzed total protein extracts with BioA-specific polyclonal antiserum. As expected, no BioA was detected in *ΔbioA* and BioA expression was not changed by atc in wt *Mtb* (Figure 4A). In *bioA* TetON-1 extracts prepared from atc-containing cultures BioA protein exceeded the wt levels, but BioA protein was not detected in extracts of cultures grown without atc. Because *bioA* TetON-1 grew, albeit more slowly, in the absence of both atc and biotin (Figure 3A), these results suggested that the amount of BioA expressed by wt is actually significantly higher than required for growth in biotin-free media.

The immunoblots also indicated that induction of BioA by atc in *bioA* TetON-1 was stronger than necessary to achieve full complementation. Seven nucleotides upstream of the *bioA* start codon, the *bioA* mRNA of *bioA* TetON-1 contains a hexamer that is perfectly complementary to the region of *Mtb*'s 16S rRNA thought to bind mRNAs during the initiation of translation. We sought to improve regulation of BioA expression by mutating this putative ribosome binding site (Shine-Dalgarno site, SD) and constructed three mutants, *bioA* TetON-2/-3/-4, in which individual nucleotides were mutated as shown in Figure 4B. In the fifth mutant, *bioA* TetON-5, all nucleotides of the SD were changed to decrease binding of the *bioA* TetON-5 mRNA to the ribosome. With atc, *bioA* TetON-2/-3/-4/-5 all expressed less BioA than *bioA* TetON-1 and the lowest amount of BioA was detected in extracts of *bioA* TetON-5 (Figure 4A). The experiments shown in Figure 4A did not allow us to measure BioA expression in the different TetON mutants without atc, but we expected that mutations in the SD also decreased the amount of BioA expressed by each mutant in the absence of atc and tested this by measuring growth without biotin.

**Figure 5:**
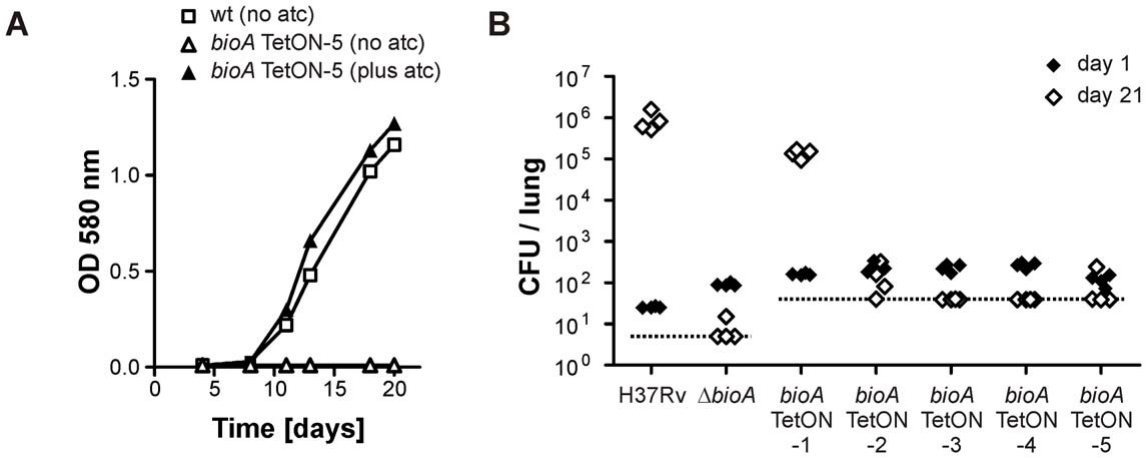
Growth of *Mtb bioA* TetON-5 in biotin free liquid media and of all mutants in mice without doxy. (A) Growth of wt and *bioA* TetON-5 in biotin-free Sauton's medium with and without atc. Squares represent data for wt, triangles represent data for ***bioA*** **TetON-5**. Open symbols indicate data from medium without atc, closed symbols represent data from medium containing atc. Data are from individual cultures and representative of at least two independent experiments. (B) Growth of wt, *ΔbioA* and the different ***bioA*** **TetON** mutants in mouse lungs. Closed symbols represent CFUs from day 1 post infection, open symbols represent data from day 21. The dotted line indicates the limit of detection (which was lower for wt and *ΔbioA* than for the TetON mutants due to the different amounts of lung homogenates that were plated). Each symbol represents CFUs obtained from one mouse.

In contrast to *bioA* TetON-1 (Figure 3A), *bioA*-TetON-2/-3/-4/-5 all failed to grow in biotin-free media lacking atc. With atc, growth of the new mutants was indistinguishable from growth of wt and from growth with biotin (Figure 5A and Figure S1). Moreover, growth of the mutants was strictly atc-dose dependent (Figure S2 and not shown). Next, we infected mice and monitored growth of *ΔbioA* and all five *bioA* TetON mutants without doxy (Figure 5B). Similar CFU counts were recovered from mouse lungs for all mutants 1 day after the aerosol infection. 21 days post infection, the CFUs recovered from the mice infected with *bioA* TetON-1 had increased from 161 (±11) to 136,800 (±31,704) whereas the CFUs of *ΔbioA* and *bioA* TetON-2/-3/-4/-5 were close to or below the limit of detection.

Together, these experiments demonstrated that (i) BioA levels in wt *Mtb* are significantly above those required for growth without exogenous biotin, (ii) efficient regulation of BioA expression by TetR and P_myc1_ *tetO* required mutation of the ribosome binding sequence, and (iii) conditionally silenced *Mtb bioA* strains TetON-2/3/4/5 could not replicate in mouse lungs in the absence of doxy.

### *BioA* is essential for persistence of *Mtb* during chronic infections

**Figure 6:**
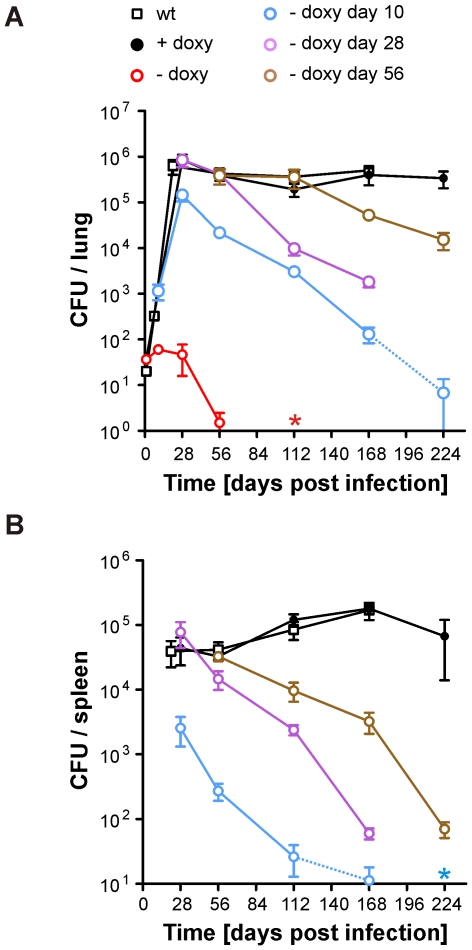
Growth and survival of *Mtb bioA* TetON-5 in mouse lungs and spleens. (A) Growth and persistence in lungs. Squares represent data for wt, circles represent data for *bioA* TetON-5. *Mtb bioA* TetON-5 was analyzed in mice that did not receive doxy (red circles), received doxy from day 1 to day 10 (blue circles), from day 1 to day 28 (purple circles), from day 1 to 56 (brown circles) or received doxy throughout the infection (black circles). Data are averages from at least four mice and two independent infections; error bars represent the standard error of the mean. The limit of detection was 4 CFUs per lung. Dotted lines end in data points for which most of the lungs contained 4 or fewer CFUs. The red asterisk indicates that no CFUs were recovered from any of the no-doxy mice 112 days post infection. (B) Persistence in spleens. The blue asterisk indicates that on day 224 no CFUs were recovered from any of the mice that received doxy up to day 10. Otherwise as described for (A).

Expression of BioA was lowest in *bioA* TetON-5 (Figure 4) suggesting that this mutant might also allow most efficient silencing of BioA without atc. We therefore selected it to determine the extent to which *Mtb* requires BioA to persist during the chronic phase of infection in a murine model of TB and silenced *bioA* transcription on days 1, 10, 28 or 56 post infection. In accordance with the infection described above (Figure 5B), *bioA* TetON-5 failed to grow in mice that never received doxy (Figure 6), with no more than 4 CFUs isolated from lungs 56 days post infection and none recovered after 112 days. In the absence of doxy, *bioA* TetON-5 thus reproduced the phenotype of *ΔbioA* (Figure 3B).

In mice receiving doxy continuously, CFUs recovered from lungs increased during approximately four weeks, were almost constant in lungs and spleens thereafter, and were very similar to CFUs of wt *Mtb* H37Rv. Deprivation of biotin after the first 10 days resulted in growth impairment of *bioA* TetON-5 in lungs and spleens and perhaps more importantly caused a subsequent decline of CFUs to less than 0.02% of the day 28 CFUs from lungs by 224 days post infection. By that time no CFUs could be recovered from the spleens of these *bioA* TetON-5 infected mice. When biotin deprivation was initiated 28 days post-infection, the CFUs in lungs decreased by more than 99% at day 168. Due to a technical problem we did not obtain data for this group of mice 224 days post infection (or the wt control). However, silencing *bioA* even later in the chronic phase of the infection, at day 56, resulted in a ∼96% reduction in bacterial numbers recovered from lungs at day 224. Histological analyses demonstrated that silencing of BioA expression 10 days post infection prevented the severe pathology caused by *bioA* TetON-5 with doxy and that inactivation of BioA at day 56 reduced lung pathology at later time points (Figure S3). In summary, these experiments demonstrated that *bioA* expression is required for *Mtb* to both establish and maintain infection in a murine model of TB.

### Quantitative analyses of the BioA levels and activities of different mutants

**Figure 7:**
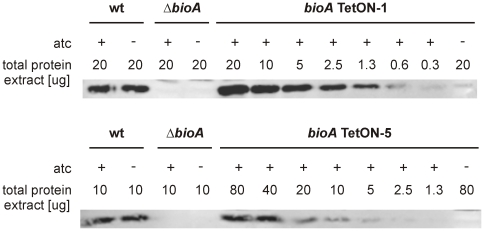
Quantitative BioA immunoblotting of *bioA* TetON-1 and *bioA* TetON-5. Immunoblots were performed as described in the main text and in the material and methods.

Our final goal in this study was to determine the minimal level of BioA expression required for *in vitro* growth and survival of *Mtb* in mice. To do so, we first analyzed immunoblots that contained serial dilutions of total protein extract for all TetON mutants. The intensity of the BioA band in these blots was measured using an Infrared Imaging System, normalized to the total protein amount loaded, and compared to wt protein extracts. Two representative immunoblots of this kind are shown in Figure 7 and the relative BioA levels measured by this approach are shown in Table 1.

**Table 1:** Relative BioA protein levels and activities.

	BioA protein*		BioA activity*	
	Plus atc	Minus atc	Plus atc	Minus atc
*Mtb ΔbioA*	<1%	<1%	<1%	<1%
*Mtb bioA* TetON-1	1,160%	7%	1,526% ± 483%	4.3% ± 2.3%
*Mtb bioA* TetON-2	230%	<1%	320% ± 22%	<1%
*Mtb bioA* TetON-3	184%	<1%	403% ± 64%	<1%
*Mtb bioA* TetON-4	14%	<1%	36% ± 4%	<1%
*Mtb bioA* TetON-5	9%	<1%	25% ± 14%	<1%

*BioA protein levels and BioA activities are expressed relative to wt *Mtb*, which was defined to express 100% BioA protein/activity. BioA activity error estimates represent standard deviations of up to four measurements.

We next developed a coupled assay to measure BioA activities in cell lysates using BioD catalyzed formation of DTB. In this assay, saturating concentrations of substrates and cofactors were added to cell lysates along with recombinant BioD to catalyze conversion of DAPA into DTB. DTB was then purified from the reaction mixture using streptavidin agarose resin and eluted into buffer by thermal treatment of the resin (Figure S4). DTB was subsequently detected by displacement of the fluorescent DTB probe *N* ^1^-[3-[2-(2-{3-[(Fluorescein-5-yl)carbonyl]aminopropoxy}ethoxy)ethoxy]propyl}dethiobiotinamide (Fl-DTB) from streptavidin resulting in an increase in the fluorescent signal. This assay proved to be extremely sensitive and enabled detection of as little as 30 femtomoles of BioA in the cell lysates. The amount of DTB produced was directly proportional to the concentration of BioA (Figure S5). For each assay a standard curve was generated by adding known amounts of DTB to *ΔbioA* lysates (Figure S6). The relative BioA activity measured by this approach is also shown in Table 1.

BioA expression levels estimated by immunoblotting were somewhat lower than those obtained by the coupled enzyme assay but generally in good agreement with the results of this BioA activity assay. Both assays demonstrated that regulation of expression of *bioA* in *bioA* TetON-1 was efficient and without atc the BioA activity of this mutant was reduced by ∼96% compared to wt *Mtb* (Table 1). For *bioA* TetON-2/3/4/5 BioA protein levels and activities were reduced by at least 99%.

## Discussion

Here, we report an *Mtb* mutant, *ΔbioA*, in which the entire open reading frame encoding DAPA synthase was deleted, to evaluate the *in vitro* and *in vivo* characteristics of *Mtb*'s BioA as a potential drug target. Deletion of *bioA* was confirmed genotypically (Figure 1C) and phenotypically in experiments showing that *ΔbioA* expressed no BioA protein (Figure 4A), had no BioA activity (Table 1), and produced decreasing amounts of biotinylated protein after transfer into biotin-free media (Figure 2C). In biotin-free medium, *ΔbioA* lost viability (Figure 2D) with kinetics similar to those observed for an *Mtb bioF* mutant [11]. The *in vitro* growth defect of *ΔbioA* could be complemented with biotin at concentrations as low as 50 nM (Figure 2B), which is at least 25-times higher than the biotin concentration in human serum [21], [22]. Growth of *ΔbioA* was also rescued with DTB, but not using KAPA as a substrate since conversion of KAPA to biotin is BioA-dependent (Figures 1A and 2A). Following aerosol infection, *ΔbioA* failed to replicate in mice and was cleared from the lungs of several mice during the first 8 weeks of the infection (Figures 3B and 5B).

Survival of an *M. smegmatis* mutant, which contains a transposon insertion in *bioA* (referred to as 272A), was impaired during stationary phase even in the presence of exogenous biotin [23]. That we did not observe such a defect for *Mtb ΔbioA* (Figure 2D) suggests that *M. smegmatis* and *Mtb* differ with respect to the conditions in which they are able to utilize exogenous biotin. However, what exactly caused the survival defect of *M. smegmatis* 272A is not entirely clear because this mutant required several vitamins to grow and it is unknown if a wt copy of *bioA* would complement its stationary phase survival defect [23]. What is clear, however, is that the growth and survival defects that *Mtb ΔbioA* displayed *in vitro* and during infections can be complemented by expression of an intact copy of *bioA* (Figures 3, 5A, 6, S1, S2, and S3). Our experiments thus demonstrate that *Mtb* cannot acquire biotin from the host and requires *de novo* biotin biosynthesis to both establish and maintain a chronic infection in a murine model of TB. This dependence on *de novo* biotin biosynthesis is likely conserved in other mycobacterial pathogens, as a biotin auxotroph of *M. marinum* was also attenuated [13].

To determine *Mtb*'s dependency on biotin synthesis over the course of infection, we constructed TetON mutants, in which transcription of *bioA* was induced with atc or doxy and repressed in the absence of both tetracycline derivatives. This allowed us to deprive *Mtb* of its ability to synthesize biotin after it had grown to more than 1,000 CFUs in both lungs and spleens or had already established a chronic infection. Irrespective of the stage of the infection at which shutdown of *bioA* was initiated, it always had a severe impact on growth and survival of *Mtb* (Figure 6). In mice that received doxycycline from day 1 to day 10, CFUs increased for approximately 18 more days. That the switch from doxy-containing to doxy-free food did not result in a more immediate impact is likely because BioA, biotin, and the biotinylated enzymes that were synthesized prior to removal of doxy have to be depleted or inactivated before survival of *Mtb* is affected. Beginning on day 28 post infection, CFUs in the lungs declined with a rate of 14% (±5%) per week (Figure 6A), which caused most mice to have less than 4 CFUs per lungs 224 days post infection. If silencing was initiated at 28 or 56 days post infection lung CFUs decreased with rates of 12% (±1%) and 10% (±1%) per week, respectively, at later time points. Silencing of *bioA* also had a drastic impact on survival of *Mtb* in mouse spleens (Figure 6B) and histological studies demonstrated that silencing *bioA* during chronic infections decreased lung pathology at later timer points (Figure S3). These data do not prove that silencing *bioA* at the later time points can actually sterilize a chronic infection in the lungs. However, once the CFUs started to decrease they continued to do so with similar kinetics irrespectively of the time point at which silencing was initiated, which suggests that sterilization would have occurred eventually. Taken together, this demonstrates that *Mtb* relies on biotin synthesis for growth and survival throughout the course of mouse infections. It furthermore indicates that the reported inactivity of amiclenomycin in *Mtb* infected mice [16], may have been due to poor pharmacokinetic behavior of this compound, a conjecture supported by our observation that amiclenomycin spontaneously decomposes through aromatization (Orisadipe, A., Boshoff, H. I., Barry, C. E., unpublished results).

We previously pointed out that genetic drug target validation studies often fall short of assessing the vulnerability of a target to partial inhibition [24]. This is relevant because chemical inactivation of a target during an infection is likely incomplete and enzymes that need to be inactivated by, for example, 80% will, on average, make better targets than those that need to be inactivated by 99% before a pathogen stops replicating. We employed two approaches, protein level and protein activity measurements, to determine the extent to which BioA needs to be inactivated before *Mtb* stops growing. In general, these two approaches gave similar results but minor discrepancies were apparent for mutants that expressed low levels of BioA in the presence of atc. This was likely due to very low signal intensities in the respective immunoblots. Both analysis methods indicated that inactivation of BioA has to reach more than 90% before growth of *Mtb* is severely affected *in vitro* or during infections. This is because *Mtb bioA* TetON-1 still grew without atc and without biotin *in vitro* and also grew without doxy in mice even though its BioA protein level and activity were reduced by more than 90% without atc/doxy. Mutants in which the expression and activity of BioA was reduced by at least 99% showed strong growth and survival defects *in vitro* and during infection.

It is impossible to compare vulnerability of BioA to incomplete inhibition to that of other potential *Mtb* drug targets because BioA is the first *Mtb* protein for which vulnerability has been measured. But studies in which vulnerability of different essential enzymes to incomplete depletion was measured have recently been published for *M. smegmatis* [25], [26]. These studies suggest that BioA might not be a highly vulnerable target because reducing expression of the RNA polymerase subunit B, RpoB, by as little as 80% was sufficient to prevent growth in *M. smegmatis* [26] and similar levels of inactivation might suffice to stop replication of *Mtb* as well. It thus seems prudent to determine if other enzymes in the biotin synthesis pathway might be more susceptible to partial inactivation than BioA. However, the reported activity of amiclenomycin against whole *Mtb* cells suggests that sufficiently potent inhibitors of BioA can be obtained and the structure of amiclenomycin provides a starting point for their design. We expect the mutants we described here to help with the development of such amiclenomycin derivatives and the identification, evaluation and development of other inhibitors of *Mtb*'s biotin metabolism, because they should be more susceptible than wt to such inhibitors.

Amiclenomycin is known to covalently inactivate BioA in the process of aromatization [27] thus leading to an unusual situation analogous to 100% enzyme inhibition. A recently appreciated variable in determining target vulnerability is the ability to identify inhibitors with long residence times such as those that form covalent linkages or that induce conformational changes upon binding their targets. In TB residence time may be correlated with *in vivo* effect [28]. Such factors would seem likely to be more important with relatively invulnerable targets such as BioA than with more vulnerable targets such as RpoB. Percent inhibition required for an effect on growth is, of course, only one factor important in determining the overall target vulnerability. Nonetheless such information offers an important new way to prioritize potential targets within a biochemical pathway and may explain some of the failure in translating highly potent inhibitors of essential enzymes into compound series with high cellular potency [29].

That so little is known about vulnerability of essential proteins to incomplete depletion is in part due to the difficulties we and others have experienced in constructing phenotypically well-regulated conditional *Mtb* knockdown mutants. Here, we overcame a key technical hurdle associated with evaluating a potential drug target *in vitro* and in mice: the leakiness of transcriptional regulatory systems, which can often prevent efficient silencing of proteins required only in small amounts, for which we developed a novel translational regulatory strategy. This may provide a generally applicable approach that will enable vulnerability to be added as a parameter of target validation. Such information should help to further focus target-based drug discovery efforts on pathways and enzymes that are essential under a variety of conditions and are susceptible to incomplete inhibition.

## Material and Methods

### Ethics statement

All procedures including animal studies were conducted following the National Institutes of Health guidelines for housing and care of laboratory animals and performed in accordance with institutional regulations after protocol review and approval by the Institutional Animal Care and Use Committee of Weill Cornell Medical College (protocol # 0802-713A, TetR-controlled gene silencing in mycobacteria during mouse infections).

### Strains, media and culture conditions

*Mtb* H37Rv was obtained from Dr. Robert North (Trudeau Institute) and grown in Sauton's liquid medium containing 0.02% tyloxapol, or in Middlebrook 7H9 liquid medium (Difco) containing 0.2% glycerol, 0.5% BSA, 0.2% dextrose, 0.085% NaCl, and 0.05% Tween 80, or on Middlebrook 7H11 agar plates containing 10% OADC supplement (Becton Dickinson) and 0.5% glycerol. When required, hygromycin B was used at a concentration of 50 or 100 µg/ml and kanamycin at a concentration of 15 µg/ml. Preparation of competent cells, electroporations, and preparation of genomic DNA were performed as described [19]. Where indicated, 7-keto-8-aminopelargonic acid (KAPA; Toronto Research Chemicals), DTB (Sigma), and biotin (Sigma) were added at a concentration of 1 µM.

### Mutant construction and southern blotting

*BioA* was deleted by homologous recombination following transduction with a derivative of the temperature sensitive mycobacteriophage phAE87 [30]. To generate this phage the 3′-portion of the *rv1567c* was amplified by PCR, digested with *Bgl*II and *Nco*I and cloned into likewise digested pJSC284 (gift of J. S. Cox) resulting in pJSC284-Rv1567c. We then amplified the *bioA* promoter region and the 5′-portion of *bioF* using primers that allowed us to fuse these two amplicons in a third PCR. The resulting PCR product (in which the *bioA* promoter region was located upstream of the 5′-portion of *bioF*) was cloned downstream of the hygromycin resistance cassette of pJSC284 using *Afl*II and *Age*I. This resulted in pJSC284-Rv1567c-bioF, which contained the complete *bioA* knockout cassette. The integrity of pJSC284-Rv1567c-bioF was confirmed by restrictions and DNA sequencing. pJSC284-Rv1567c-bioF was used to construct a *bioA* knockout phage as described [31]. Hygromycin-resistant transductants of *Mtb* were selected on 7H11 agar and obtained after 3 weeks of incubation at 37°C. Mutants were analyzed by PCR and Southern blotting. For Southern blotting genomic DNA was digested with *Dra*III and analyzed with a probe specific for *bioF*. Hybridization and detection were carried out with an ECL chemoluminescent detection system (GE Healthcare) as instructed by the manufacturer. *BioA*-TetON mutants were obtained by transformation of *ΔbioA* with expression plasmids that integrate into the attachment site of the mycobacteriophage L5, contain *bioA* downstream of P_myc1_ *tetO* [19] and also contained *tetR*#2 [20]. These plasmids were constructed using gateway recombination as described [32].

### Immunoblots

Cell lysates were prepared by bead-beating cell pellets in lysis buffer (25 mM Tris HCl [pH 7.6], 1 mM EDTA, and 1 mM PMSF) and sterilized by passage through a 0.22 µm Spin-X filter (Costar). For the detection of biotinylated proteins, 15 µg cell lysates were subjected to SDS-PAGE, followed by transfer to a nitrocellulose membrane. Biotin-specific mouse serum (Sigma) and DlaT-specific rabbit serum [33] were used at 1∶1,000 dilutions in blocking buffer. For the detection of BioA proteins, cell lysates were subjected to SDS-PAGE and blots were probed with BioA-specific rabbit serum (1∶1,000) generated against purified BioA protein. Goat anti-mouse and goat anti-rabbit (LI-COR Biosciences) were diluted 1∶10,000 in Odyssey blocking buffer. Blots were developed using the Odyssey Infrared Imaging System (LI-COR Biosciences).

### BioA activity assay

Bacteria were grown in at least 500 ml Sauton's media containing 1 µM biotin in the presence or absence of atc. Once the culture reached an OD_580_ of ∼ 1.0, bacteria were harvested by centrifugation, washed, and resuspended in lysis buffer (100 mM bicine pH 8.6, 50 mM NaHCO_3_, 1 mM MgCl_2_, 0.0025% igepal CA-630, and protease inhibitor cocktail tablet [Roche]). Cell free lysates were prepared by beating the solution with 0.1 mm Zirconia/Silica beads (BioSpec Products, Inc) three times at 6000 rpm for 50 sec at 4°C, followed by 10 min centrifugation at 13000 rpm, 4°C and sterilized by passage through a 0.22 µm Spin-X filter (Costar). BioA activity in the lysates was quantified using a coupled assay employing BioD to generate DTB, which was subsequently detected in a fluorescence displacement assay. BioA and BioD were expressed and purified as described (Wilson, D. J. et al., submitted) Biotin and biotinylated proteins were removed from cell free lysates by the addition of 100 µL streptavidin agarose resin (Thermo Scientific 20349) that had been pre-washed with PBS to remove storage buffer.

Samples were incubated for 30 min at 4°C, spun at 1000 × g for 1 min, and the biotin free supernatant was retrieved. Reactions were then prepared in duplicate in 500 µL total volume and contained 200 µL biotin-free *Mtb* lysate (1–20 mg protein/mL), 12.5 µM KAPA, 5 mM SAM, 1 mM TCEP, 5 mM ATP, 200 µM PLP in reaction buffer (100 mM bicine pH 8.6, 50 mM NaHCO_3_, 1 mM MgCl_2_, 0.0025% igepal CA-630). Reactions were initiated by the addition of 320 nM BioD and incubated at 37°C for 16 hours. Control reactions were made by omitting BioD or freezing control reactions at −80°C. Both controls yielded identical results. DTB formed was removed from reactions by the addition of 30 µL of streptavidin agarose resin followed by incubation at 4°C for 30 min. Resin was pelleted at 1000 × g for 1 min and the resin was washed with 300 µL reaction buffer plus 10 mM MgCl_2_. DTB was removed from the resin by incubating the beads with 47 µL of water for 5 min at 110°C. The free DTB was detected by transferring 45 µL of the reaction mixture into a black 384 well plate (corning 3575) and adding *N* ^1^-[3-[2-(2-{3-[(Fluorescein-5-yl)carbonyl]aminopropoxy}ethoxy)ethoxy]propyl}dethiobiotinamide (Fl-DTB) [34] and streptavidin (Sigma S4762) providing a final concentration of 20 nM and 185 nM, respectively in a total of 50 µL. Plates were incubated for 10 min and read on a molecular devices M5e multi mode plate reader using excitation 485, emission 530, and cutoff 530 nm. To quantitate the amount of DTB produced a standard curve was produced by adding known quantities of DTB to reactions containing lysate from the Δ*bioA* mutant (see Figure S6 for a representative standard curve). The curve was fit to equation (1) using GraphPad Prism (version 4):
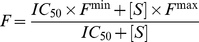
(1)where *F* is the fluorescence intensity at DTB concentration [*S*], *F*_max_ and *F*_min_ are the maximum and minimum fluorescence signals respectively and IC_50_ is the concentration of DTB that produces 50% *F*_max_. Fluorescence was then converted back into DTB concentration using equation (2).
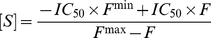
(2)


Experiments to determine that the amount of DTB produced was directly proportional to the concentration of BioA were performed by adding 0, 0.5, 1,0, and 2.0 nM recombinant BioA to the standard reaction above using lysate from the Δ*bioA* mutant (2 mg/mL). The amount of DTB produced per concentration of BioA was fit with linear regression analysis using Prism (Figure S5).

### Mouse infections

8–10 week old C57BL/6 mice were purchased from Jackson Laboratory and infected with the indicated *Mtb* strains by aerosol as described [35]. When indicated, mice received doxycycline containing mouse chow (2,000 ppm; Research Diets). Serial dilutions of organ homogenates from four mice per data point were plated onto 7H11 agar plates to quantify CFUs. When histopathology analyses were performed, the upper left lobes of infected lungs were fixed in 10% buffered formalin, sectioned and stained with hematoxylin and eosin (Laboratory of Comparative Pathology at Memorial Sloan-Kettering Cancer Center).

## Supporting Information

Figure S1 ***In vitro*** **growth of** ***Mtb*** ***bioA*** **TetON mutants.** Growth of wt and *bioA* TetON-1/2/3/4/5 in biotin-free Sauton's medium with and without atc and in biotin-containing Sauton's medium. Data are from individual cultures and representative of at least two independent experiments.
(TIF)

Figure S2 **Atc dose-responsive growth of** ***bioA*** **TetON-5.** Growth of *bioA* TetON-5 in biotin-free Sauton's with the indicated atc concentrations was analyzed in 96-well microtiter plates. The culture volume per well was 100 µL and growth was measured after incubation at 37°C for 20 days.
(TIF)

Figure S3 **Histopathology of mouse lungs infected with** ***Mtb bioA*** **TetON-5.** Each panel shows sections of entire lobes from 4 different mice on the right and a smaller section from one lung at larger magnification on the left.
(TIF)

Figure S4 **Schematic diagram of the BioA activity assay.** DTB is generated from KAPA using BioA (either recombinant protein or *Mtb* protein extract) and recombinant BioD in a reaction containing saturating concentrations of all substrates (SAM, ATP, HCO_3_) and required cofactors (PLP, Mg^2+^). DTB is subsequently detected by displacement of a fluorescently labeled DTB analog (Fl-DTB, shown as either black/quenched or green/fluorescent) from streptavidin (grey oval) resulting in an increase in the fluorescent signal. Assay details are described in the materials and methods.
(TIF)

Figure S5 **Standard curve of recombinant BioA activity.** Each reaction contained 200 µL TB Δ*bioA* lysate (2 mg/mL), 0.5, 1, or 2 nM BioA, 12.5 µM KAPA, 5 mM SAM, 1 mM TCEP, 5 mM ATP, 200 µM PLP and 320 nM BioD in reaction buffer (100 mM Bicine pH 8.6, 50 mM NaHCO_3_, 1 mM MgCl_2_, 0.0025% igepal CA-630) and was processed as described in the materials and methods.
(TIF)

Figure S6 **Standard curve of displacement of Fl-DTB by DTB.** Each reaction contained 200 µL TB Δ*bioA* lysate (4 mg/mL), 12.5 µM KAPA, 5 mM SAM, 1 mM TCEP, 5 mM ATP, 200 µM PLP and 0–500 nM dethiobiotin in reaction buffer (100 mM Bicine pH 8.6, 50 mM NaHCO_3_, 1 mM MgCl_2_, 0.0025% igepal CA-630) and was processed as described in the materials and methods.
(TIF)
